# tDCS modulates speech perception and production in second language learners

**DOI:** 10.1038/s41598-022-20512-0

**Published:** 2022-09-28

**Authors:** Katy Borodkin, Tamar Gassner, Hadeel Ershaid, Noam Amir

**Affiliations:** Department of Communication Disorders, Sackler Faculty of Medicine, Tel Aviv University, P.O. Box 39040, 6997801 Tel Aviv, Israel

**Keywords:** Neuroscience, Psychology

## Abstract

Accurate identification and pronunciation of nonnative speech sounds can be particularly challenging for adult language learners. The current study tested the effects of a brief musical training combined with transcranial direct current stimulation (tDCS) on speech perception and production in a second language (L2). The sample comprised 36 native Hebrew speakers, aged 18–38, who studied English as L2 in a formal setting and had little musical training. Training encompassed musical perception tasks with feedback (i.e., timbre, duration, and tonal memory) and concurrent tDCS applied over the left posterior auditory-related cortex (including posterior superior temporal gyrus and planum temporale). Participants were randomly assigned to anodal or sham stimulation. Musical perception, L2 speech perception (measured by a categorical AXB discrimination task) and speech production (measured by a speech imitation task) were tested before and after training. There were no tDCS-dependent effects on musical perception post-training. However, only participants who received active stimulation showed increased accuracy of L2 phoneme discrimination and greater change in the acoustic properties of L2 speech sound production (i.e., second formant frequency in vowels and center of gravity in consonants). The results of this study suggest neuromodulation can facilitate the processing of nonnative speech sounds in adult learners.

Adult second language (L2) learners often find it difficult to perceive and pronounce accurately speech sound contrasts that are phonemic in L2 but not in their native language (e.g., ship *vs* sheep or think *vs* sink for some English language learners)^1^. It has been suggested that the inaccuracies sometimes arise because nonnative sounds are assimilated into native language phonetic categories^2,3^. These difficulties reduce listening comprehension^4^ and contribute to nonnative-sounding speech production^5^. Foreign accent often decreases speech intelligibility and comprehensibility^6^ and negatively affects listener’s perception of the speaker^7^. Previous studies suggest that musical expertise may alleviate some of the difficulties in both perception and production of nonnative speech sounds^8,9^. However, musical expertise requires years of intensive musical practice. In the current study, we tested whether a brief musical training enhanced by non-invasive brain stimulation can also facilitate perception and production of L2 speech sound contrasts.

Musical and speech sounds share acoustical properties that convey information, including pitch, timing, and timbre. Pitch, the perceptual equivalent of fundamental frequency, refers to the organization of sound on an ordered scale (low *vs* high); timing refers to the temporal features of the sound (e.g., its onset and duration), and timbre, which has both temporal and spectral features, refers to the quality of the sound^10^. These commonalities and the expertise with musical sounds that musicians acquire with training have led researchers to hypothesize that musical training may improve speech perception.

Consistent with this hypothesis, it has been demonstrated that musician children were more able to discriminate between L1 sounds that were manipulated acoustically based on their temporal features (i.e., vowel duration and consonant voice onset time) and spectral features (i.e., fundamental frequency of the vowel)^11^. Moreover, musicians were more sensitive than nonmusicians when processing a nonnative consonant contrast, which was evident in their categorization performance and in brain electrical activity^12^. In another study, nonnative musicians showed similar neural response to spectral information in L2 vowels as native speakers. More specifically, the amplitude of brainstem response to vowel formants in English was higher in both native English speakers and English as L2 speakers who were musicians compared to English as L2 speakers with no musical experience^9^. Furthermore, “musical ear” (an innate aptitude for music) was reported to be related to perception of L2 sounds in persons with no or little musical training^13^. Finally, the causative role of musical training in enhancing speech perception was demonstrated in a longitudinal study, in which a one-year musical training (but not painting training) resulted in enhanced processing of the temporal features of speech sounds, as reflected in syllabic duration and voice onset time for consonants^14^.

One way to explain the improvement in speech perception induced by musical training is by assuming shared neural resources. According to this hypothesis, processing efficiency in brain regions that are responsive to both music and speech sounds improves as a consequence of intensive musical training, which in turn enhances the efficiency of speech processing^15^. Alternatively, the transfer account postulates that long-term musical training results in functional adaptation of speech-specialized brain regions. That is, brain regions dedicated to speech processing are also available for musical processing in musicians, which allows for a better perception of speech sounds^16,17^.

While it is difficult at present to determine the contribution of each of these mechanisms to the superior speech perception in musicians, converging evidence suggests that the left posterior auditory-related cortex, including the posterior superior temporal gyrus (pSTG) and the planum temporale (PT), supports both music and speech processing at the level of single sounds. The left PT has been long recognized as the “temporal speech region” since the discovery of its morphological leftward asymmetry^18^. This same region was associated in a later study with general auditory functions: Increased leftward asymmetry of the left PT was reported in musicians with absolute pitch compared to musicians with relative pitch or nonmusicians^19^. The recruitment of the bilateral PT in various tasks has led researchers to conclude it is a “computational hub” in charge of spectro-temporal decoding of the acoustic signal^20^. Furthermore, it was observed that the structural and the functional characteristics of the left PT might change as a result of long-term musical training, which in turn may benefit processing of speech sounds. Increased activity in and cortical surface area of the left PT was associated with better perception of speech sounds in musicians compared to nonmusicians^21,22^. Similarly, better recognition of musical and speech tokens in musicians was related to enhanced activation of the left pSTG^17^.

Numerous studies have reported that musical aptitude and musical training improve not only speech perception but also L2 speech production^8,13,23,24^. For instance, among Japanese-English speakers who acquired English after the age of 11 years, higher musical aptitude was correlated with better L2 production skills, as assessed by native speakers^13^. Likewise, productions of nonnative Mandarin tones were rated more native-like in musicians compared to non-musicians, and the acoustic properties of the speech signal (i.e., fundamental frequency) were more similar to native speakers in musicians than non-musicians^8^. Musical practice also enhances the outcomes of L2 pronunciation training. In one study involving Polish speakers of English as L2, musicians produced more native-like vowels (as measured by formant values) than non-musicians did after a two-semester accent training^25^. Among American English speakers who received a single training session on producing Hindi consonants, greater training benefits were observed in musicians than in non-musicians^26^.

The positive effects of musical aptitude and expertise on both L2 speech perception and production are consistent with previous findings of a perception-production link in L2 learners^27^. According to the Speech Learning Model, this relationship is unidirectional in that accurate perception of L2 speech sounds is a necessary precursor to their accurate production^2^. The more recent revised Speech Learning Model suggests a bidirectional relationship, in which perception and production co-evolve without precedence^28^. Other scholars propose a developmental perspective within which the strength and the quality of the perception-production link changes with advances in L2 learning^29^. Extending the accounts to the effects of musical training on L2 speech perception and production, the Speech Learning Model may predict that musical training increases the sensitivity of the left posterior auditory-related cortex (including the pSTG and the PT) to perceive L2 phoneme contrasts, thus facilitating their accurate production. The revised Speech Learning Model may predict more direct effects of musical training on speech production via the posterior part of the left PT (also dubbed area Spt). This region is active in both speech perception and production^30–32^, suggesting it serves as an interface between auditory and motor systems^33^. The Spt is not exclusively dedicated to speech processing; it responds as strongly to listening and humming covertly musical melodies^34^. Musicians show increased activity in the left Spt while covertly performing music^35^ and increased connectivity between this region and motor areas in a resting state^36^. The increase in the efficiency of the sensorimotor integration circuitry following musical training may thus positively affect both the ability to perceive and pronounce L2 speech sounds.

A small but growing body of research explored the effects of transcranial direct current stimulation (tDCS) on perception and production of speech sounds. tDCS is a non-invasive brain stimulation technique, in which a low-intensity electrical current is applied over the scalp using a positive (anode) and a negative (cathode) electrode. Anodal tDCS is believed to have an excitatory effect by temporarily facilitating depolarization of the neurons in the targeted cortical region, which promotes long-term potentiation and cortical plasticity^37^. Previous research has demonstrated that tDCS over the left temporal lobe and the temporo-parietal region modulates electrophysiological responses to auditory stimuli^38^ and changes the regional cortical neurotransmitter balance^39^. It has been further shown that anodal stimulation of the left auditory cortex enhances spectral processing^40^ and bilateral anodal tDCS increases temporal processing^41^ of non-speech sounds. Beyond the low-level acoustic processing, tDCS over the auditory cortex was shown to modulate speech perception. For example, tDCS resulted in a more precise categorization of consonant–vowel syllables on a voice onset time continuum accompanied by changes in the auditory cortex reactivity^42^.

In the domain of speech production, tDCS was most often applied over the frontal regions of the left hemisphere, which improved speech motor learning in healthy individuals^43,44^ and facilitated speech production in neurodegenerative apraxia of speech^45^. Two studies also demonstrated beneficial effects of anodal tDCS placed more posteriorly on motor speech learning and rehabilitation. Specifically, stimulation over the left auditory cortex improved speech production accuracy in post-stroke apraxia of speech^46^ and simulation over the inferior parietal lobe enhanced speech motor learning in healthy individuals^47^.

The current study explored whether a brief musical training combined with non-invasive brain stimulation can improve perception and production of L2 sounds in adults with little musical training, resembling the positive effects of long-term, intensive musical training. The musical training included discrimination between musical tones based on timbre or duration, which engages spectral and temporal processing. Since speech perception also relies on fine-grained distinction between spectral and temporal features of speech sounds, we reasoned such training could be beneficial for processing of L2 sounds as well. Musical short-term memory was also trained, as it is related to verbal short-term memory^48^, which predicts perception^49^ and production^50^ of L2 speech sounds; furthermore, short-term memory in both domains is subserved by a shared network of regions including the left PT^51^. Anodal or sham tDCS (control condition) was applied over the left posterior auditory-related cortex (including the pSTG and PT). We hypothesized that active stimulation over an area that supports both music and speech processing combined with brief musical training would improve the perception of musical tones and of L2 sounds, as measured by accuracy and speed of response. We also expected the pronunciation of L2 sounds to improve, as indexed by the acoustic analysis of their spectral and temporal properties.

## Method

### Participants

The sample included 36 right-handed participants (19 men), aged 18–38 years. They were native speakers of Hebrew who were first exposed to English as L2 at elementary school (mean age of acquisition = 8.54 years) and learned the language in a school setting. Participants were recruited if they knew no languages other than Hebrew and English, had no significant musical training, and were not music professionals. In line with safety guidelines for tDCS studies^52^, participants were also excluded if they had a medically diagnosed neurological or psychiatric condition, epilepsy or a single seizure episode, scalp or skin condition, or any metallic implants. Pregnant women were also not included in the study. These exclusion criteria were verified using a translated to Hebrew version of a tDCS screening questionnaire^52^.

Half of the participants were randomly assigned to the active stimulation group (*n* = 18, 9 men) and the other half—to the sham stimulation group (*n* = 18, 10 men). As Supplementary Table S1 indicates, the groups were matched on multiple background variables, including age, years of education, handedness, age of acquisition of English as L2, current exposure to English, English proficiency, the degree of nonnative accent, previous musical training, and frequency of listening to music (*p* > 0.10). Information on background variables was collected using a self-reported questionnaire, which was devised for this study. Handedness was evaluated using the Hebrew version of the Edinburgh Handedness Inventory^53^.

Twenty participants were unpaid volunteers, and the remaining 16 were paid 120 NIS (approximately $35) for their time. There was an equal number of paid and unpaid participants in the two experimental groups.

### Musical training

The musical training in this study was comprised of three auditory discrimination tasks from the Seashore test^54^ of musical aptitude, which evaluated the perception of timbre, duration, and tonal memory. In the timbre task, participants had to decide whether two tones in a pair were identical or different. In the duration task, they were asked to decide whether the second tone in a pair was shorter or longer than the first tone. In the tonal memory subtest, participants listened to pairs of short, almost identical tunes (tune length ranged from 3 to 5 tones) and identified the tone that was different in the second tune. The timbre and duration tasks each included 30 tone pairs, and the tonal memory task included 18 pairs of tunes. The pairs of each task were arranged in order of increasing difficulty.

Musical training was delivered using a Lenovo ThinkPad E-560 laptop, screen size 15.6", E-prime software, v. 2.0^55^, and supra-aural Sony MDR-CD380 stereo headphones. A digital version of the Seashore test items was used with permission from Paulo Esquef^56^. A trial in each training task started from a short instruction, which was presented on the screen for 2000 ms, followed by a plus sign as a fixation mark for 700 ms, which in turn was followed by a pair of tones or tunes, depending on the task. Participants were given 5000 ms (in the timbre and duration tasks) or 10,000 ms (in the tonal memory task) from first tone onset to respond. A longer time window for response in the tonal memory task was provided to account for item length. The trial ended with written feedback on the response correctness and speed of the trial and the overall accumulated percent of correct responses across trials. The training was completed in approximately 20 min.

### Transcranial direct current stimulation

Stimulation was administered using a battery driven, constant current stimulator (neuroConn DC stimulator plus, Incl GmbH). The anodal electrode covered in a normal saline-soaked, synthetic sponge was placed over CP5 (i.e., left posterior STG and the PT), according to the 10–20 international system for EEG electrode placement. The reference electrode was positioned over the contralateral supraorbital region. The size of both electrodes was 5 cm × 7 cm. In the active stimulation group, the current was given for 20 min at 1.5 mA intensity. In the sham stimulation group, the stimulation was ramped up for the first 30 s, remained active for 30 s, and then was ramped down for another 30 s. This procedure has been shown to keep participants blind of the respective stimulation condition^57^. The study had a double-blind design: neither the participants, nor the experimenters were aware of group assignment. To ensure experimenter blindness, electrodes were removed only 20 min after the stimulation was completed, when the electrodes cooled down. A safety questionnaire^58^ was administered at the end of the stimulation. Participants were asked to report about feelings of headache, difficulty concentrating, change in mood or vision, fatigue, and skin sensations (yes or no questions). They were also asked to guess which stimulation they received, active or sham.

### Pre- and post-training assessment

Pre- and post-training tasks were administered using the same equipment and software as described for musical training.

#### Musical perception

Timbre, duration, and tonal memory subtests of the Seashore test^54^ were used to evaluate musical perception. Half of the items in each subtest appeared during the training session, and the remaining half were new. This design allowed us to study both training and generalization effects. The paradigm was identical to that of training, but no feedback was provided. Accuracy and reaction time were calculated separately for the training and generalization sets of each subtest.

#### L2 speech perception

L2 speech perception was examined using a categorical AXB discrimination task of phoneme contrasts in English. There were three vowel contrasts (i.e., /i/-/I/, /ɛ/-/æ/, and /ʌ/-/ɑ/), which are nonnative to Hebrew, which has only five vowels, including /i/, /e/, /a/, /o/, and /u/. In addition, there were four consonant contrasts (i.e., /z/-/ð/, /d/-/ð/, /s/-/θ/, and /t/-/θ/), also nonnative to Hebrew. Hebrew has the sounds /z/, /d/, /s/, and /t/, but not the dental fricatives /ð/ and /θ/. Each phoneme in a contrast had five word tokens, resulting in 35 minimal pairs and a total of 70 words (see Table 1 for examples). The words were recorded with a sampling rate of 44,100 Hz and a bit depth of 16 bit/sample, as uttered by three female speakers, all of which were native speakers of general American English (raised in Illinois).

**Table 1:** Examples of stimuli in the categorical AXB discrimination task.

Contrast	Word tokens
**Vowel contrasts**	
/i/-/I/	Sheep-ship
/ɛ/-/æ/	Bed-bad
/ʌ/-/ɑ/	Bus-boss
**Consonant contrasts**	
/z/-/ð/	Zen-then
/d/-/ð/	Day-they
/s/-/θ/	Sick-thick
/t/-/θ/	Tie-thigh

Each trial consisted of three words pronounced by three different speakers. The first and the third word were always different. The words were uttered sequentially, at a pace of one word per second. The computer screen remained blank while the words of a triplet were played out. An exclamation mark appeared next and remained on the screen for 2500 ms, during which time participants had to indicate, by pressing the appropriate key, whether the second word was similar to the first or the third word.

Each word in a minimal pair served as a middle word in one of the triplets. Such triplets were presented in separate blocks, each containing 35 triplets, so that in one block the first word of a pair was placed in the middle and in the other—the second word of a pair (e.g., *sheep*, *sheep*, and *ship* in one block and *sheep*, *ship*, and *ship* in another). The block order was randomized across participants. The three speakers and the place in the sequence of the target word (i.e., first or last) were also randomly ordered across participants. The task started with 10 training words that were different from the task words. The task was completed in approximately 10 min.

#### L2 speech production

Speech production in English was evaluated using a speech imitation task, in which participants were asked to listen to and repeat words in English. The stimuli list contained the 70 words of the AXB discrimination task. Each trial contained a single word, which was simultaneously presented auditorily via headphones and visually on the laptop screen. Such multimodal elicitation was previously suggested to facilitate imitation in L2 speakers^1^. No time limit was imposed on responses.

The stimuli were presented in three blocks, each containing the entire set of 70 words uttered by one of the native English speakers. Thus, each word was repeated three times, to increase the number of tokens for the acoustic analysis. The blocks and the stimuli in each block were presented in a random order across participants. The task started with 10 training words, which were not included in the stimuli list of the task. Participants completed the task within 10 min on average.

The responses were recorded using a Shure MX153 headworn microphone, a Behringer XENYX 302 USB audio interface to amplify and digitize the signal, and Praat software^59^ and later analyzed acoustically.

Spectral (first and second formants for vowels and center of gravity for consonants) and temporal (vowel and consonant duration) properties of the L2 target speech sounds were analyzed (the speech sounds that established minimal pairs in the AXB discrimination task). The acoustic analysis included two stages: segmentation and annotation, followed by extraction of various temporal and spectral characteristics. The analysis was based mainly on manual extraction, with some degree of manually supervised automatic extraction, as detailed below.

All recordings were segmented manually via visual inspection of the signals alongside their spectrograms. This was performed in Praat software, using the annotation module, by trained research assistants. A random sample of the segmentations was verified by one of the authors, and whenever systematic errors were uncovered, the research assistant was instructed to redo the segmentation. The segments were annotated according to the target consonants and syllables, which were determined by the experimental procedure, and did not involve any perceptual judgments at this stage. Segment durations, used later in the statistical analysis, were derived directly from the Praat annotation files (“TextGrids”).

**Figure 1:**
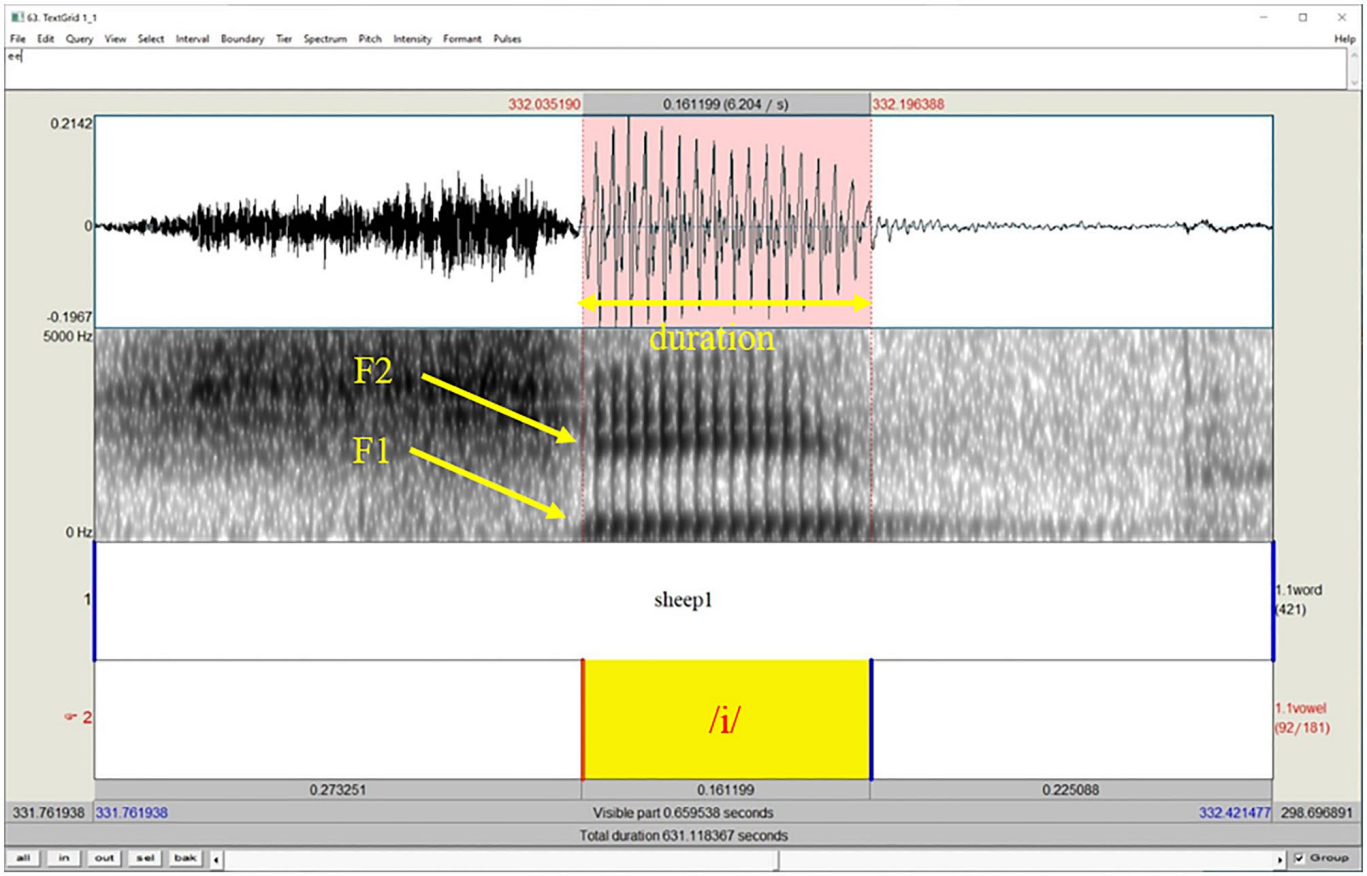
Example of a spectrogram showing the spectral and the temporal properties of the vowel /i/ embedded in “sheep”.

For vowel formant extraction, initial calculation of the first two formants (F1 and F2) was performed automatically by custom written Matlab routines, using the same procedure as Praat software. Men’s vowels were downsampled to 8 kHz and women’s were downsampled to 11 kHz. Linear Predictive Coding (LPC) was then applied, using an order of 8 for men and 10 for women. The resulting poles were retained only if they were above 90 Hz and having bandwidths less than 400 Hz. Similar to previous reports, this analysis was not reliable enough, giving some false values due to various particularities of the recorded voices^60,61^. It was therefore decided to perform visual inspection of the formant values. To this end, once more custom written software written in Matlab was used. The software incorporated a Graphic User Interface (GUI), which could rapidly cycle through the extracted vowel signals, and present the formant tracks superimposed on a wide-band spectrogram. In cases where the spectrogram and the formant tracks were in disagreement, the user could adjust the LPC order to improve this agreement. The entire corpus of 6480 vowels was scanned and corrected where necessary in this manner, by trained research assistants. This was also verified on a random sample by one of the authors. The final values of F1 and F2 for each vowel were taken from the central 30 ms of the vowel (see Fig. 1 for an illustration of the two formants and the duration of a vowel on a spectrogram).

In order to perform a unified analysis on men’s and women’s data, formant values for men were subsequently normalized. The ratio of mean F1 for women, divided by mean F1 for men was found to be 1.183, with the corresponding value for F2 being 1.172. Therefore, men’s F1 values were all multiplied by 1.183, and men’s F2 values were all multiplied by 1.172. Thereafter the statistical analysis was performed on all participants as a single group.

Consonants were analyzed for duration, as were the vowels. In addition, the spectral Center of Gravity (COG), which is the power-weighted average frequency of a spectrum, was also calculated^62^. COG has a tunable parameter “p”, which was set to 2 in the present study. It was computed here by custom written Matlab scripts, using the same formula as Praat. Overall, 8,640 consonants were analyzed. Because some of the consonants were grossly mispronounced (e.g., /z/ was produced instead of /ð/), a perceptual judgement of all of the consonants was carried out by two native American English speakers. In cases of disagreement, a third judge was added. Only consonants that were perceived by two judges as matching the experimental targets were included in the analyses. As a result, 57 consonants were discarded (0.7% of the data). This procedure aimed to reduce the amount of extraneous variability in the acoustic measurements.

### Procedure

The study was conducted in a quiet room, in two sessions. During the first session, participants received an explanation about the study, signed a consent form, and filled out questionnaires. Informed consent was obtained from all participants. Next, they completed the three assessment tasks, in the following order: the three subtests of the Seashore test^54^, imitation task, and categorical AXB discrimination task. This order (perception-production-perception task) was established to reduce monotonicity in task requirements and minimize fatigue effects. The second session was conducted 3–5 days after the first one. It began with the musical training combined with tDCS, following which the tDCS side effects questionnaire^58^ was administered. Participants then completed again the three assessment tasks, which were delivered in the order of the first session. Each session lasted about an hour.

### Ethical statement

This study was approved by the Institutional Review Board of Tel Aviv University (approval # 0000718-1), and all methods were performed in accordance with the Declaration of Helsinki. Informed consent was obtained from all participants.

## Results

Mean raw data at baseline is presented in Supplementary Tables S2–S5. As these tables demonstrate, accuracy on the timbre subtest of the musical perception test and on three of the contrasts of the phoneme discrimination task (i.e., /ʌ/–/ɑ/, /z/–/ð/, and /s/–/θ/) was significantly lower in the active stimulation group compared to the sham stimulation group. Statistical analyses were thus conducted on difference scores, which were calculated by subtracting post-training scores from pre-training scores for all dependent variables. This procedure was previously recommended in case of baseline imbalance, which may occur as a result of random fluctuations despite random group assignment. Difference scores perform better than other methods in terms of Type 1 error rates when there are baseline difference and imperfect reliability of scores (such as the case of empirical data collection)^63^; they have the least amount of bias compared to other methods when the pre-test group difference is moderate (point biserial correlation between group and pre-test scores equals approximately to *r* = 0.40, as in this study)^64^, and they are reliable when the correlation between the pre- and post-training scores is low^65^ (*r* < 0.20 in this study). Effects of stimulation group were analyzed using one-way MANOVAs after verifying that the respective dependent variables were inter-correlated (*r* >|.30|).

### Musical perception

Accuracy on the trained and untrained sets of the Seashore subtests was evaluated in two separate one-way MANOVAs with group (active, sham) as the independent variable and difference scores on timbre, duration, and tonal memory subtests as the dependent variables. Similar analyses were conducted on reaction speed. None of the analyses yielded a significant multivariate group difference (all *F*s < 1), suggesting tDCS had no effect on performance.

### L2 speech perception

**Figure 2:**
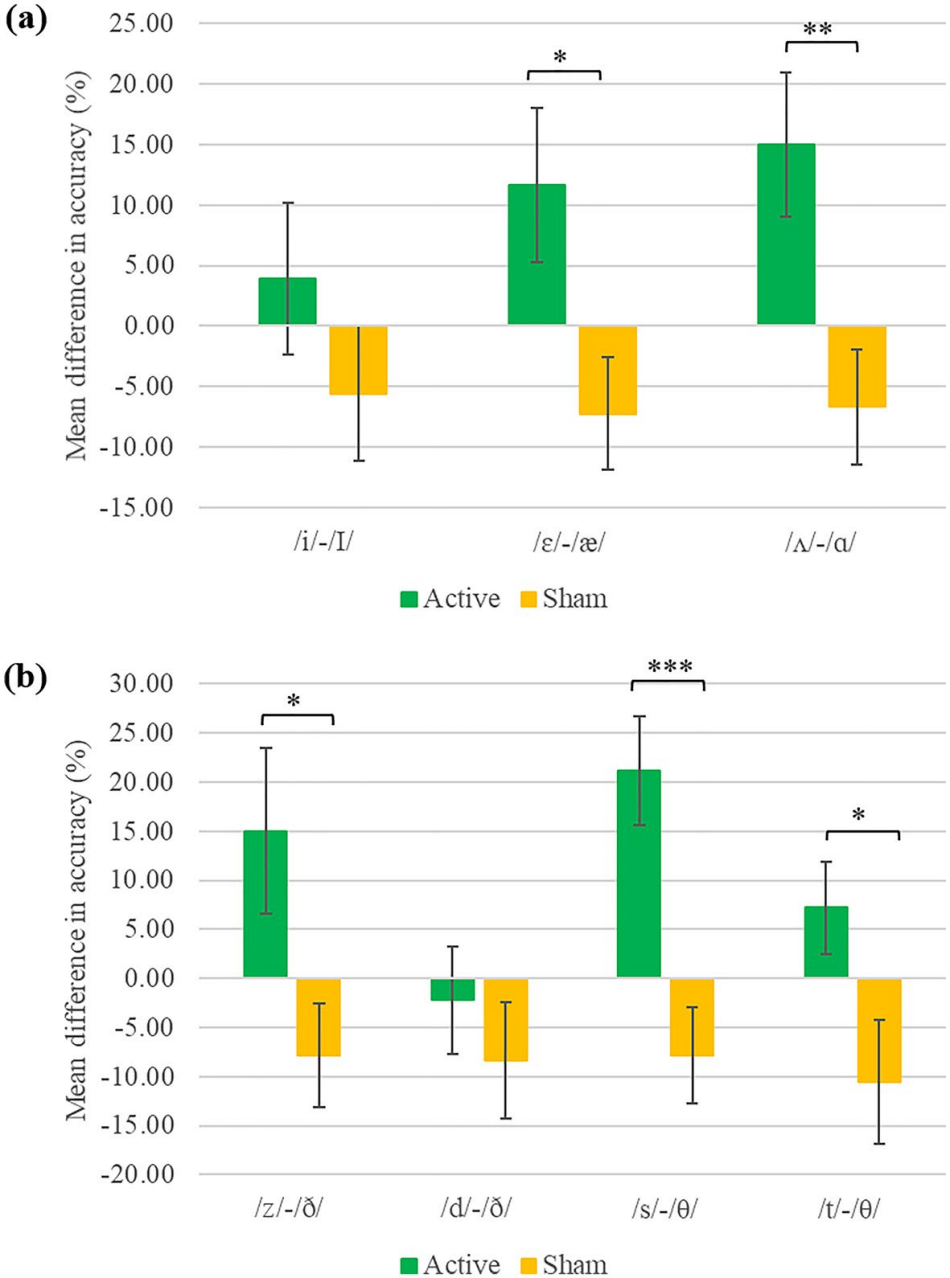
Difference scores in discrimination accuracy (%) of phoneme contrasts following active and sham stimulation. (**a**) Represents vowel contrasts and (**b**) consonant contrasts. Mean difference was calculated by subtracting post-training scores from pre-training scores. Error bars represent standard error of the mean. *p < .05, **p < .01, ***p < .001.

A one-way MANOVA with group (active, sham) as a between-subject variable was conducted on the difference scores in discrimination accuracy of vowel contrasts, which yielded a significant multivariate effect of group, *F*(3,32) = 2.90, *p* = 0.05, Wilk’s λ = 0.79. Follow-up univariate ANOVAs suggested the effect was significant for the /ɛ/-/æ/ contrast, *F*(1,34) = 5.74, *p* = 0.02, and the /ʌ/-/ɑ/ contrast, *F*(1,34) = 8.05, *p* = 0.008. As Fig. 2a indicates, improvement in both contrasts was observed in the active stimulation group only.

Another one-way MANOVA on difference scores in discrimination accuracy of consonant contrasts also resulted in a significant multivariate effect of group, *F*(4,31) = 4.68, *p* = 0.005, Wilk’s λ = 0.62. Follow-up univariate ANOVAs suggested the effect was significant for the /z/-/ð/ contrast, *F*(1,34) = 5.23, *p* = 0.03, the /s/-/θ/ contrast, *F*(1,34) = 15.30, *p* < 0.001, and the /t/-/θ/ contrast, *F*(1,34) = 5.13, *p* = 0.03. As shown in Fig. 2b, discrimination accuracy for each of the three contrasts increased in the active stimulation group only.

Since the difference scores in the two groups were in most cases in the opposite direction (i.e., accuracy increased in the active group and decreased in the sham group), we also conducted a series of one-sample *t*-tests (one-tailed), in which mean difference score in each group was compared to 0. These comparisons yielded a significant result for two vowel contrasts (/ɛ/-/æ/: *t*(17) = 1.83, *p* = 0.042, and /ʌ/-/ɑ/: *t*(17) = 2.52, *p* = 0.011) and two consonant contrasts (/z/-/ð/: *t*(17) = 1.78, *p* = 0.047, and s/-/θ/: *t*(17) = 3.81, *p* < 0.001) in the active group. All of the comparisons in the sham group were nonsignificant (*p* > 0.05), suggesting the observed increase post-training following active stimulation reflected a real improvement while the observed decrease following sham stimulation resulted from a random fluctuation.

There were no significant multivariate or univariate group effects in difference scores of reaction times for vowel or consonant contrasts (*p* > 0.40).

### L2 speech production

Three one-way MANOVAs with group (active, sham) as the independent variable were conducted on the difference scores of F1, F2, and duration of the six vowels of the imitation task (i.e., /i/, /I/, /ɛ/, /æ/, /ʌ/, and /ɑ/). None of the multivariate or univariate group effects were significant (*p* > 0.10). A visual inspection of the individual data indicated that the groups might differ in the distance from baseline in the post-training utterances, irrespective of the direction of the change.

**Figure 3:**
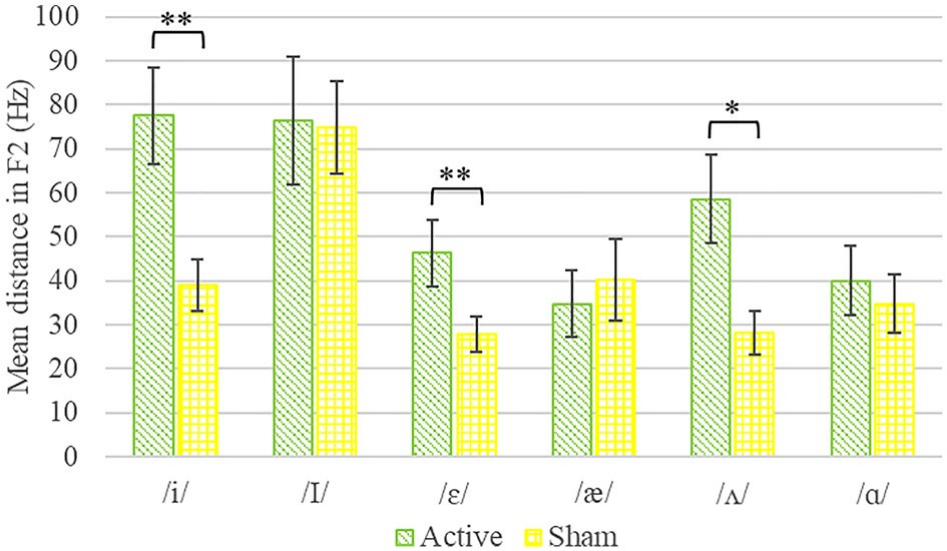
Mean distance scores of F2 frequency of vowels in the active and sham stimulation groups. Mean distance scores were calculated as the absolute value of the difference between post- and pre-training values. Error bars represent standard error of the mean. *p < .05, **p ≤ .01.

To explore this possibility, we calculated distance measures as the absolute value of the difference between post- and pre-training values of F1 and F2 frequencies and duration of each vowel. A one-way MANOVA with group as an independent variable on the distance scores of F1 values yielded a non-significant result, *F*(6,29) < 1. A similar MANOVA on the distance scores of F2 values resulted in a significant multivariate effect of group, *F*(6,29) = 2.62, *p* = 0.04, Wilk’s λ = 0.65. In follow-up univariate ANOVAs, this group difference was evident in word tokens containing /i/, *F*(1,34) = 9.64, *p* = 0.004, /ʌ/, *F*(1,34) = 7.39, *p* = 0.01, and /ɛ/, *F*(1,34) = 4.66, *p* = 0.04. For each vowel, the distance between F2 values post- relative to pre-training was larger in the active stimulation compared to the sham stimulation group (for descriptive statistics, see Fig. 3). Lastly, a one-way MANOVA on the distance scores of vowel duration did not yield a significant multivariate effect of group, *F*(6,29) < 1.

**Figure 4:**
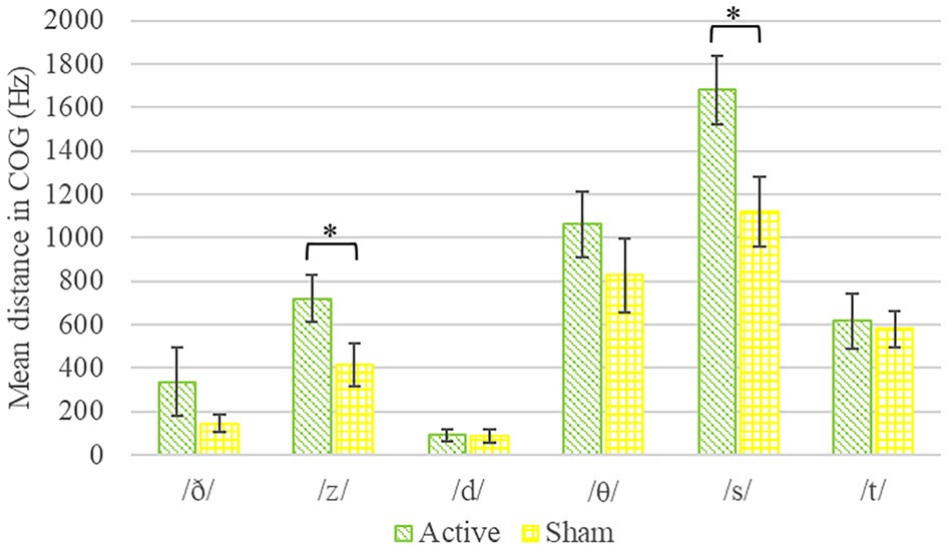
Mean distance scores of COG (center of gravity) values of consonants in the active and sham stimulation groups. Mean distance scores were calculated as the absolute value of the difference between post- and pre-training values. Error bars represent standard error of the mean. *p < .05.

We next analyzed the acoustic parameters of the six consonants of the study, including /ð/, /z/, /d/, /θ/, /s/, and /t/. One-way MANOVAs with group as a between-subject independent variable on the difference scores of COG and (separately) of duration yielded no significant multivariate or univariate effects (*F*s < 1). We then calculated distance scores to measure changes in COG and duration values irrespective of the direction of change for each consonant. A one-way MANOVA on the distance scores in COG values resulted in a significant multivariate group effect, *F*(6,29) = 2.38, *p* = 0.05, Wilk’s λ = 0.67. Follow-up univariate ANOVAs showed this group difference was significant for utterances containing /z/, *F*(1,34) = 4.38, *p* = 0.04, and /s/, *F*(1,34) = 6.12, *p* = 0.02. The active stimulation group had larger distance scores of COG for both consonants relative to the sham stimulation group (see Fig. 4 for means and standard errors). A one-way MANOVA on the distance scores of duration values yielded a nonsignificant multivariate group effect, *F*(6,29) < 1.

### Safety questionnaire

tDCS-related complaints (e.g., headache, fatigue, and skin sensations) were infrequent overall and comparable across the study groups, *p* > 0.10. Eleven participants in the active stimulation group and nine participants in the sham stimulation group correctly guessed their group assignment. A chi-square test of independence yielded a non-significant result, χ^2^(1) = 0.45, *p* = 0.50, confirming participants were unaware of group assignment.

## Discussion

The difference scores on the musical perception tests were similar across the experimental groups, suggesting there was no tDCS effect. However, the stimulation seems to have affected L2 speech perception and production, as was observed in the increased accuracy of phoneme discrimination post-training, and the greater change in the spectral characteristics of vowel and consonant production in the active stimulation group. While the left posterior superior temporal cortex was previously indicated in both music and speech processing^17,21,22,30–32^, the region may have been more engaged in the latter in this study because of differences in previous experience. Participants in our study may have been accustomed to discriminating and pronouncing English language sounds, with which they were familiar, as English was their second language. They may have had much less experience with discriminating musical tones and tunes, as they had little musical training. It is thus possible that the musical perception tasks were more difficult than the speech processing tasks (as supported by accuracy data in Supplementary Tables S2 and S3), which may have resulted in task-dependent tDCS effects. Alternatively, the involvement of the left posterior auditory-related cortex may be dependent on musical expertise. It was shown, for example, that listening to piano music involves the left PT in skilled musicians and its right hemisphere homologue in nonmusicians^66^. Anodal tDCS over the right posterior superior temporal gyrus may thus be considered in future research as the target region of neuromodulation in individuals with no musical training.

tDCS-induced improvement in the accuracy of L2 phoneme discrimination in this study is consistent with previous neuroimaging research highlighting the involvement of the left posterior superior temporal gyrus and the left PT in phoneme processing and phoneme discrimination in particular in the native^17,21,22,67,68^ and nonnative languages^69,70^. We used difference scores to study the improvement in speech perception, as recommended for our data^63,64^. We followed up the group comparisons with further analyses showing that the mean difference score across phoneme contrasts was significantly different from 0 in the active group but not in the sham stimulation group, providing support that the change post-training was not a result of random fluctuations only in the former. Nevertheless, it is still possible that the change in the accuracy of phoneme discrimination was partially accounted for by baseline differences, because they were present in three of the four contrasts that showed improvement in the active stimulation group. We thus treat these findings with caution. We also note that for a full interpretation of the improvement in the perception task, data from native English speakers is needed.

Turning to L2 speech production data, the effects of the combined training were not as straightforward. The acoustic properties of L2 speech sounds did not change in a consistent direction in either group. However, participants in the active stimulation group increased the distance from baseline in the spectral features of their utterances post-training. In other words, they attempted more than participants in the sham stimulation group to change their utterances following tDCS, although the direction of the change was not consistent across participants. These results are in line with previous research showing faster and greater changes in speech perception than production following perception training^27,29^. The fact that most speech sounds that underwent change post-stimulation (i.e., /i/, /ɛ/, /ʌ/, /z/, and /s/) comprised the contrasts for which discrimination accuracy also increased (i.e., /ɛ/-/æ/, /ʌ/-/ɑ/, /z/-/ð/, and /s/-/θ/) suggests that changes in speech production may have followed improvement in speech perception. Consistent with the Speech Learning Model^2^, according to which perception of nonnative sounds restricts their production, participants in the active stimulation group may have acquired a more precise auditory representation of the phonemes, which resulted in better identification of the inaccuracies in their production and hence, in greater attempts to alter the utterances. The revised Speech Learning Model, on the other hand, claims that L2 perception and production change in parallel^28^. According to this account, active tDCS over the left posterior auditory-related cortex may have facilitated the establishment of more accurate auditory representations of speech sounds and also elicited spectral changes in the utterances via its effects on sensorimotor integration. Previous research has demonstrated that the left PT supports the transformation of the perceived speech sounds to their phonemic and motor representations for articulation in both native^30,31^ and nonnative speech^71,72^. It is thus possible that exposure to the English language sounds uttered by native speakers during the imitation task immediately following tDCS over this region increased the efficiency of sensorimotor integration resulting in increased attempts to bring closer the auditory and the motor representations of L2 sounds. The attempts were inconsistent, thus only observable in the distance but not the difference scores, perhaps because their amount in the imitation task was too small to lead to consistent changes.

A few further findings of the speech imitation task are of note. We will limit the discussion to vowels, because their acoustic properties have been previously studied in much more detail compared to consonants. First, it is interesting that the participants in the active stimulation group attempted to change F2 values of only some but not all of the vowels (i.e., /i/, /ɛ/, and /ʌ/). To further explore this finding, we compared formant values obtained at baseline in this study with previously reported formant values of native English speakers^73^. We should note, however, that a better comparison would involve data collected specifically for this study from native English speakers pronouncing the same words as the L2 speakers. As Table 2 suggests, participants in the active stimulation group attempted changing F2 frequencies in the vowels of each pair that were most distant from native speakers’ pronunciation. Thus, tDCS facilitated the identification of the largest deviation from the L1 norm and elicited adjustments in their pronunciation. The table also contains mean F2 frequencies of three Hebrew vowels^74^ that are similar to the English vowels. Inspection of the values suggests little evidence of assimilation-related phenomena or that changes consistently occurred in the vowels that were most distant from the native language sounds^2^. Second, the changes following active stimulation occurred in the spectral but not the temporal properties of L2 vowels. It has been previously suggested that L2 learners of English sometimes proceed from attending to the temporal properties of L2 sounds to attending to their spectral properties with increased proficiency, as native English speakers do^75,76^. Since participants in this study have been exposed to English for at least 10 years, they may be considered intermediate-advanced learners, which may explain their focus on the spectral properties of English vowels and consonants. Finally, the attempts at change were observable only in F2 frequencies, which may be explained by Arslan and Hansen’s finding that the midfrequency range (1500–2500 Hz, which is F2 frequency range) is the most sensitive frequency band to nonnative speaker pronunciation variations^77^. This is because minor alterations of the tongue movement can cause large shifts in this frequency band, while large changes in the F1 frequency band can only be observed following changes in the overall shape of the vocal tract. Given that participants in this study had previous experience with English vowels and that they were attempting to make subtle adjustments to their productions to approximate those of native speakers, it is plausible these attempts were most detectible in the F2 frequency band.

**Table 2:** Mean F2 frequency of the American English vowels included in the study and of similar vowels in Hebrew.

	Hebrew vowels					
	/i/		/e/		/a/	
L1 speakers^a^	2489		2073		1473	

	American English vowels					
	/i/	/I/	/ɛ/	/æ/	/ʌ/	/ɑ/
L1 speakers^b^	2730	2398	2234	2030	1396	1250
L2 speakers (active)^c^	**2588**	2313	**2143**	2007	**1614**	1382
L2 speakers (sham)^c^	2582	2331	2118	1983	1586	1401

Numbers in bold show a significant change following tDCS in the active stimulation group.

^a–b^Values are from^74^ and^73^, respectively, after multiplying male values by 1.172 and averaging across men and women for easier comparison with our values.

^c^Values recorded pre-training.

To summarize, while this study has shown little effect of a brief musical training together with anodal tDCS over the left auditory-related cortex on musical skills, there was a positive impact on L2 speech perception and production. The changes in speech perception and production after a single session that lasted less than 30 min are particularly significant when evaluated against previous perception training studies, which typically included multiple sessions delivered across 20 days on average^27^. To our knowledge, this is the first study to explore the potential of neuromodulation to facilitate speech processing in a L2, and as such, it lays the ground for future research to develop accent reduction programs that combine novel brain stimulation techniques. Remediation of nonnative accent is an important endeavor for many individuals, since accented speech reduces listener’s understanding of the message, often causing communication breakdowns^78^, and negatively affects social evaluations, such that speakers with a nonnative accent are perceived as less intelligent, successful, trustworthy^7^, and judged as less employable^79^. It would be interesting to further explore our findings in order to define the conditions that will lead to optimal outcomes in terms of L2 speech processing, such as stimulating additional brain regions (e.g., the motor and the premotor cortex), increasing the amount of training sessions, and combining tDCS with speech perception or speech production training.

## Supplementary Information

Supplementary Information.

## Data Availability

The datasets used and/or analysed during the current study available from the corresponding author on reasonable request.
