# Supplementary material for: tDCS modulates speech perception and production in second language learners
Source: Sci Rep. 2022 Sep 28;12:16212. doi: 10.1038/s41598-022-20512-0 (PMC9519965; doi:10.1038/s41598-022-20512-0)
Supplement: Supplementary file 1 — Supplementary Information. [file 41598_2022_20512_MOESM1_ESM.docx]

**tDCS modulates speech perception and production**

**in second language learners**

Katy Borodkin*, Tamar Gassner, Hadeel Ershaid, and Noam Amir

**Supplementary Table S1**. Means and Standard Deviations (in Parentheses) of Background Variables in the Experimental Groups.

|  | Active (n =18) | Sham (n = 18) |
| --- | --- | --- |
|  | M (SD) | M (SD) |
| Age (years) | 28.17 (5.85) | 27.94 (5.45) |
| Education (years) | 14.94 (2.15) | 14.44 (2.20) |
| Handedness^a^ | 90.00 (8.40) | 93.61 (7.44) |
| English as L2 |  |  |
| Age of acquisition (years) | 8.53 (1.27) | 8.56 (1.29) |
| Current exposure (% of time) | 13.72 (13.80) | 11.44 (9.76) |
| Proficiency^b^ | 7.14 (1.12) | 6.86 (1.72) |
| Nonnative accent^c^ | 5.72 (2.21) | 5.44 (2.28) |
| Musical training (years) | 1.17 (1.71) | 0.64 (1.14) |
| Frequency of listening to music^d^ | 3.83 (0.38) | 3.83 (0.51) |

Note. The groups did not significantly differ on any of the variables (p > .10).

^a^Scores on the Edinburgh Handedness Inventory range from 0 to 100, with higher scores indicating greater right hand preference.

^b^Scale ranges from 0 to 10, with higher ranks indicating greater proficiency.

^c^Scale ranges from 0 to 10, with higher ranks indicating greater nonnative accent.

^d^Scale ranges from 0 to 4, with higher ranks indicating more frequent listening to music.

**Supplementary Table S2**. Performance at Baseline on the Subtests of the Seashore Test in the Active and Sham Simulation Groups.

|  | Accuracy (%) | | Reaction Time (ms) | |
| --- | --- | --- | --- | --- |
|  | Active | Sham | Active | Sham |
|  | M (SE) | M (SE) | M (SE) | M (SE) |
| Trained Set |  |  |  |  |
| Timbre | 57.78 (3.62)_a_ | 69.63 (4.04)_b_ | 3204 (97) | 3056 (49) |
| Duration | 72.59 (5.55) | 80.37 (2.93) | 2533 (86) | 2417 (67) |
| Tonal memory | 53.71 (4.65) | 54.44 (4.11) | 6138 (124) | 6125 (68) |
| Untrained Set |  |  |  |  |
| Timbre | 65.56 (4.49) | 70.00 (4.71) | 3169 (132) | 3064 (49) |
| Duration | 74.07 (5.14) | 71.48 (2.27) | 2351 (65) | 2458 (50) |
| Tonal Memory | 51.11 (5.73) | 51.85 (4.80) | 5881 (97) | 5851 (128) |

Note. Percentage of correct responses and reaction times for correct responses were calculated separately for each of the three subtests (timbre, duration, and tonal memory) of the trained and the untrained set. Reaction times more than 2 SD above or below the subtest mean of an individual participant were excluded.

Means with different subscript letters differ statistically (p < .05).

**Supplementary Table S3**. Performance at Baseline on the Phoneme Discrimination Task in the Active and Sham Simulation Groups.

|  | Accuracy (%) | | Reaction Time (ms) | |
| --- | --- | --- | --- | --- |
|  | Active | Sham | Active | Sham |
|  | M (SE) | M (SE) | M (SE) | M (SE) |
| Vowel contrast | | | | |
| /i/ – /I/ | 75.00 (21.76) | 78.33 (16.18) | 718.68 (56.31) | 625.57 (53.77) |
| /ɛ/ – /æ/ | 71.67 (24.07) | 84.44 (15.80) | 724.17 (58) | 619.88 (45.10) |
| /ʌ/ – /ɑ/ | 51.67 (17.00)_a_ | 77.78 (17.00)_b_ | 703.65 (61.62) | 651.06 (60.69) |
| Consonant contrast | | | | |
| /z/ – /ð/ | 65.56 (30.34)_a_ | 85.00 (14.25)_b_ | 649.38 (35.73) | 621.21 (61.13) |
| /d/ – /ð/ | 76.67 (20.58) | 82.78 (16.02) | 683.74 (65.55) | 562.53 (59.28) |
| /s/ – /θ/ | 67.78 (27.13)_a_ | 85.56 (12.47)_b_ | 594.14 (27.08) | 599.66 (56.11) |
| /t/ – /θ/ | 73.89 (21.18) | 81.11 (11.32) | 663.67 (41.99) | 574.15 (49.95) |

Note. Percentage of correct responses and reaction times for correct responses were calculated separately for each phoneme contrast. Reaction times to vowel and consonant contrasts were excluded if they were more than 2 SD above or below their respective means of an individual participant.

Means with different subscript letters differ statistically (p < .05).

**Supplementary Table S4**. Acoustic Properties of Vowels at Baseline in the Speech Imitation Task in the Active and Sham Simulation Groups.

|  | F1 (Hz) | | F2 (Hz) | | Duration (ms) | |
| --- | --- | --- | --- | --- | --- | --- |
|  | Active | Sham | Active | Sham | Active | Sham |
|  | M (SE) | M (SE) | M (SE) | M (SE) | M (SE) | M (SE) |
| /i/ | 379 (13) | 375 (10) | 2588 (37) | 2582 (47) | 175 (8) | 173 (7) |
| /I/ | 446 (12) | 445 (9) | 2313 (35) | 2331 (55) | 142 (7) | 139 (7) |
| /ɛ/ | 632 (17) | 626 (12) | 2143 (37) | 2118 (31) | 219 (6) | 209 (8) |
| /æ/ | 774 (17) | 778 (21) | 2007 (34) | 1983 (28) | 271 (10) | 272 (10) |
| /ʌ/ | 789 (27) | 767 (19) | 1614 (24) | 1586 (29) | 150 (8) | 141 (7) |
| /ɑ/ | 751 (29) | 765 (19) | 1382 (18) | 1401 (26) | 193 (9) | 187 (8) |

Note. F1, F2 frequencies, and duration values were calculated as a mean of the five word tokens representing the vowel. The value of each word token was calculated as a mean of the three attempts to produce it.

**Supplementary Table S5**. Acoustic Properties of the Consonants at Baseline in the Speech Imitation Task in the Active and Sham Simulation Groups.

|  | COG (Hz) | | Duration (ms) | |
| --- | --- | --- | --- | --- |
|  | Active | Sham | Active | Sham |
|  | M (SE) | M (SE) | M (SE) | M (SE) |
| /ð/ | 395 (57) | 336 (55) | 130 (6) | 145 (7) |
| /z/ | 883 (94) | 875 (203) | 159 (5) | 175 (7) |
| /d/ | 302 (35) | 272 (18) | 94 (6) | 112 (7) |
| /θ/ | 1965 (372) | 1372 (275) | 180 (9) | 196 (8) |
| /s/ | 5701 (439) | 5179 (423) | 231 (8) | 254 (10) |
| /t/ | 1262 (201) | 1360 (222) | 162 (6) | 173 (9) |

Note. Center of gravity (COG; Hz) and duration (ms) were computed based on the three attempts to utter a consonant collapsed across the five word tokens of that consonant. Values of /ð/ and /θ/ were each collapsed across the 10 word tokens containing these phonemes.
